# *Treponema pallidum* Protein Tp0136 Induces Spheroidization of Vascular Endothelial Cells, Resulting in Widened Intercellular Junctions and Enhanced Vascular Permeability

**DOI:** 10.1002/smsc.202500046

**Published:** 2025-05-06

**Authors:** Yu Lin, Xi Luo, Xu Shen, Xiao‐Lin Fu, Li‐Rong Lin, Tian‐Ci Yang

**Affiliations:** ^1^ Center of Clinical Laboratory School of Medicine Zhongshan Hospital Xiamen University Xiamen University Xiamen Fujian 361000 P. R. China; ^2^ Institute of Infectious Disease School of Medicine Xiamen University Xiamen Fujian 361000 P. R. China; ^3^ Xiamen Clinical Laboratory Quality Control Center Xiamen Fujian 361000 P. R. China; ^4^ Hospital‐acquired Infection Control Department Zhongshan Hospital Xiamen University Xiamen Fujian 361000 P. R. China

**Keywords:** alternative splicing, cell spheroidization, permeability, *Treponema pallidum* protein Tp0136, vascular endothelial cells

## Abstract

The *Treponema pallidum* membrane protein Tp0136 facilitates *Treponema pallidum* dissemination, and the permeability of vasculature is intricately linked to the density of the vascular endothelial barrier, which is strongly associated with the morphology of vascular endothelial cells. In this study, utilizing the approach of inoculating Tp0136 recombinant protein into the skin lesions of rabbits infected with *Treponema pallidum*, it was observed that the Tp0136 recombinant protein induced spheroidization of vascular endothelial cells and enlargement of intercellular junctions. By high‐throughput RNA sequencing, the upregulation of the RNA‐binding protein cysteine‐ and glycine‐rich protein 1 (CSRP1) is identified, which modulated the alternative splicing of exon 19 of myosin X (MYO10), which in turn downregulated the expression of MYO10, ultimately inducing morphological spheroidization in vascular endothelial cells. Using CSRP1‐specific shRNA to knock down CSRP1 or using the alternative splicing inhibitor, the spheroidized vascular endothelial cells revert to a flattened state, suggesting that Tp0136 regulates the alternative splicing of MYO10 through CSRP1, leading to a downregulation of MYO10, followed by the spheroidization of vascular endothelial cells and an enlargement of intercellular junctions. These findings contribute to elucidating a mechanism underlying the dissemination of *Treponema pallidum*.

## Graphical abstract

*Treponema pallidum* protein Tp0136 enhances vascular permeability by upregulating CSRP1, which induces MYO10 exon 19 skipping, reducing MYO10 expression and triggering endothelial spheroidization. This study reveals a novel RNA splicing‐mediated dissemination mechanism, proposing CSRP1/MYO10 as therapeutic targets to limit bacterial dissemination in syphilis.

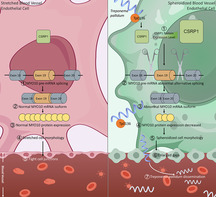

## 1 Introduction

The clandestine dissemination of *Treponema pallidum*, which is the causative agent of syphilis, takes place covertly within the human organism.^[^ ^1^ ^]^ Prior research has revealed that *Treponema pallidum* promote vascular permeability, thereby facilitating the dissemination of the pathogen throughout the bloodstream.^[^ ^2^, ^3^, ^4^ ^]^ Our previous researches have provided evidences that the *Treponema pallidum* membrane protein Tp0136 plays a crucial role in the infection process of *Treponema pallidum*. Specifically, Tp0136 has been found to activate the PI3K‐AKT pathway in endothelial cells, thereby facilitating cell migration and promoting angiogenesis. This mechanism ultimately aids in the dissemination of *Treponema pallidum*.^[^ ^5^ ^]^ However, whether Tp0136 directly induces morphological alterations in vascular endothelial cells—such as spheroidization and intercellular junction widening—through dysregulation of cytoskeletal regulatory proteins (e.g., myosins) and the underlying molecular mechanisms driving these changes remain unexplored. Nevertheless, the precise mechanism responsible for its propagation remains elusive.

Numerous studies have demonstrated a correlation between alterations in vascular permeability and the density of endothelial cell arrangement comprising the blood‐tissue barrier.^[^ ^6^, ^7^, ^8^ ^]^ The morphology of vascular endothelial cells plays a direct role in regulating the density of the blood‐tissue barrier.^[^ ^9^, ^10^ ^]^ The regulation of endothelial barrier permeability during microbial infection and dissemination is significantly impacted by morpho‐maintenance proteins, which assume a pivotal role.^[^ ^11^, ^12^, ^13^ ^]^ Myosin plays an irreplaceable role in maintaining cell morphology by regulating the contraction and extension of cytoskeletal proteins.^[^ ^14^ ^]^ A multitude of pathogenic microbes have been observed to disrupt the cytoskeletal organization of host vascular endothelial cells via obstructing the myosin synthesis, such as *Toxoplasma gondii* and *Shigella flexneri*,^[^ ^15^, ^16^ ^]^ thereby facilitating their dissemination throughout the organism.^[^ ^17^, ^18^, ^19^ ^]^ *Treponema pallidum* protein Tp0136 has been observed to stimulate the migration of fibroblast cells,^[^ ^20^ ^]^ which is accompanied by notable alterations in cell morphology. However, it remains unresolved regarding whether Tp0136 is responsible for morphological alterations through interfering the synthesis of myosin(s) in vascular endothelial cells, as well as the underlying mechanism that drives these changes.

In the present study, the vascular structure of syphilis rabbit skin lesions was scanned in the presence of the Tp0136 recombinant protein. The impact of Tp0136 protein on parameters of vascular endothelial cell line human microvascular endothelial cells was measured using an in vitro 3D blood vessel model, and the permeability of these vessels was assessed. High‐throughput RNA‐seq was utilized to analyze the alteration of the transcriptional level in mRNA expression and alternative splicing of vascular endothelial cells after exposure to Tp0136 protein. The following findings confirmed that Tp0136 protein regulates the alternative splicing of MYO10 through CSRP1, leading to a downregulation of MYO10, and induces spheroidization of vascular endothelial cells, resulting in widened intracellular junctions and enhanced vascular permeability. This finding provides a fresh perspective for further exploration of the dissemination mechanisms of *Treponema pallidum*.

## 2 Results

### 2.1 Tp0136 Spheroidized the Vascular Endothelial Cells, Enlarged the Intercellular Junctions, and Facilitated the Permeability

**Figure 1:**
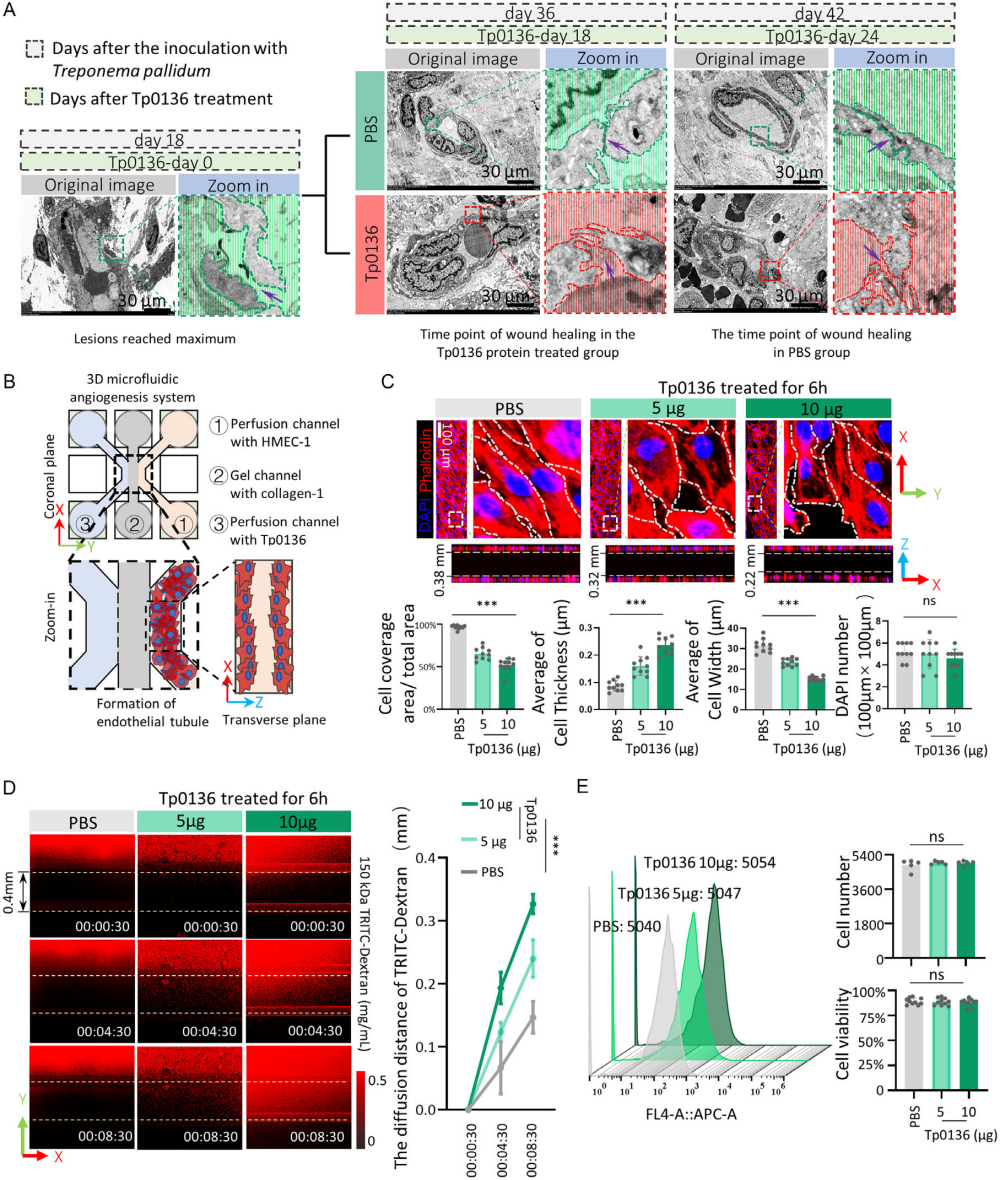
Tp0136 induces spheroidization of vascular endothelial cells, expands intercellular junctions, and enhances permeability. A) Effect of Tp0136 protein on the morphologies and intercellular junctions of vascular endothelial cells in the skin lesions of syphilitic rabbits with Scanning Electrical Microscopy. The gray bars represent the days after infection with *Treponema pallidum*. The green bars represent the days since Tp0136 protein injection. The intracellular junction gaps were indicated by purple arrows. Scale bars = 30 μm. B) Schematic drawing for 3D microfluidic angiogenesis system of angiogenesis processed by Tp0136. C) Growth of HMEC‐1 cells in a 3D microfluidic angiogenesis system. Upper: Photographs of the cells in the perfusion channel with different concentrations of Tp0136 in X‐Y panel and X‐Z panel. PBS was the negative control. Scale bars = 100 μm. Lower: The ratio of cell coverages in X‐Y panel, average of cell thickness, and average of cell width in X‐Z panel along with the rising concentration of Tp0136, and cell number calculated by DAPI number. The values represent the mean ± SD of triplicates and are representative of three independent experiments. ***, *P* < 0.001. D) Effect of Tp0136 on vascular permeability detected using 3D angiogenesis analysis. The red fluorescent images after 0 and 8 min after the addition of the TRITC‐Dextran solutions. Time is indicated in hour:minute:second. E) Cell number counted by flow cytometry, and viability of HMEC‐1 evaluated by CCK‐8 assay in 3D‐microfluid system.

To investigate the role of Tp0136 in the blood vessels, the electron microscope photographs of blood vessels in the skin lesions of syphilitic rabbit model were collected, referencing our previous study.^[^ ^5^ ^]^ Briefly, we collected the lesions at the peak of ulceration (day 18 of infection with *Treponema pallidum*) and inoculated the remaining lesions with Tp0136 protein (marked as Tp0136‐day 0). The Tp0136 protein was then inoculated every three days with PBS as a control. Then, skin lesions were collected on the 36th (marked as Tp0136‐day 18) and 42nd (marked as Tp0136‐day 24) days, and along with the lesions from the Tp0136‐day 0, these lesions were scanned using electron microscopy. It showed that, on Tp0136‐day 0, the perivascular morphologies in both the PBS group and the 10 μg Tp0136 protein group were indistinct, with zoomed‐in electron microscopy scanning revealing large gaps in the junctions of endothelial cells, which was consistent with the morphological characteristics of blood vessels around the wound of other infectious diseases.^[^ ^21^ ^]^ Subsequently, as the duration of Tp0136 protein stimulation increased, the clarity of blood vessel boundaries in both groups improved by the Tp0136‐day 18 and Tp0136‐day 24, relative to observations made on the Tp0136‐day 0. High‐magnification scanning electron micrographs revealed a progressive reduction in the gaps between vascular endothelial cells in both groups. However, the intracellular gaps between endothelial cells in the Tp0136 protein‐stimulated group remained unclosed, whereas the endothelial connections in the PBS control group had fully closed by the Tp0136‐day 18 and Tp0136‐day 24 (the intracellular junction gaps were point‐out by purple arrows). Notably, it was observed that under the exposure of Tp0136 protein, the morphology of vascular endothelial cells seemed more spherical compared to the vascular endothelial cells in the PBS group at the same time points (**Figure** **1**A). These in vivo vascular results suggested that Tp0136 protein delayed the closure of gaps between vascular endothelial cells and may promote the spheroidization of vascular endothelial cells.

To further evaluate the impact of Tp0136 on cell morphology, we utilized a 3D microfluidic blood vessel system^[^ ^22^ ^]^ to culture human microvascular endothelial cells (HMEC‐1) (Figure 1B), aiming to replicate the blood vascular structure observed in vivo. After the cells covered the lumen for 4 days, PBS and Tp0136 protein were exposed to the cells for 6 h. In the Tp0136 group, a gradual decrease in the cell coverage rate was observed as the concentration of Tp0136 protein increased (*P* < 0.001), which was 75% of cell coverage rate in cells treated with 5 μg Tp0136 protein, and declined to 50% in 10 μg of Tp0136 in X‐Y panel. Conversely, in the PBS group, the cell arrangement exhibited a dense pattern, with a cell coverage reaching 100%. Furthermore, in the X‐Z plane, the lumen diameter decreased as the Tp0136 concentration increased, with the vascular endothelial cells on the lumen wall growing as a monolayer, indicating an increase in the thickness of the vascular endothelial cells, which is a characteristic of cell spheroidization (*P* < 0.001). Concurrently, with the elevation of Tp0136 concentration, the intercellular space between vascular endothelial cells widened (*P* < 0.001, Figure 1C). Then, the permeability was measured with TRITC‐Dextran,^[^ ^23^ ^]^ which was labeled in red under the confocal microscope. It showed that the diffusion rate of TRTC‐Dextran in the Tp0136 group was significantly higher compared to the PBS group. Furthermore, treatment of vasculature‐like structures with 10 μg of Tp0136 protein resulted in a diffusion distance of 0.4 mm within 8 min, whereas a Tp0136 concentration of 5 μg limited the diffusion to 0.22 mm. These findings suggested a positive correlation between Tp0136 concentration and diffusion rate, indicating that higher concentrations of Tp0136 enhanced the diffusion rate (Figure 1D). To rule out the possibility that the increased permeability observed in the 3D microfluidic system following Tp0136 treatment is attributable to a decrease in the quantity or viability of vascular endothelial cells, we prepared a single‐cell suspension from the cellular enzymes within the 3D microfluidic system. We then quantified the number of HMEC‐1 cells under both PBS and Tp0136 treatment conditions using flow cytometry. The results indicated no significant difference in cell numbers between the Tp0136 treatment group and the PBS control group (*P* > 0.05, Figure 1E). This finding was corroborated by fluorescence microscopy, which also revealed no significant difference in the number of DAPI‐stained cells within the same field of view (*P* > 0.05, Figure 1C). Furthermore, the viability of HMEC‐1 cells was assessed via the cell counting kit (CCK‐8) assay in the 3D microfluidic system. We observed no significant difference in cell viability between the Tp0136 and PBS groups (*P* > 0.05, Figure 1E), suggesting that the increase in vascular permeability induced by the Tp0136 protein is not due to a reduction in cell number or viability. Above all, these results indicated that Tp0136 contributes to spheroidizing vascular endothelial cells, expanding intercellular junctions, and facilitating the permeability both in vivo and in vitro.

Student's *t* test or nonparametric rank‐sum test was used to compare data between two groups. The values represent the mean ± SD of triplicates and are representative of three independent experiments. ***, *P* < 0.001.

### 2.2 Tp0136 Upregulated the Expression of Cysteine and CSRP1 to Enlarge the Intercellular Junctions in HMEC‐1 Cell Line

**Figure 2:**
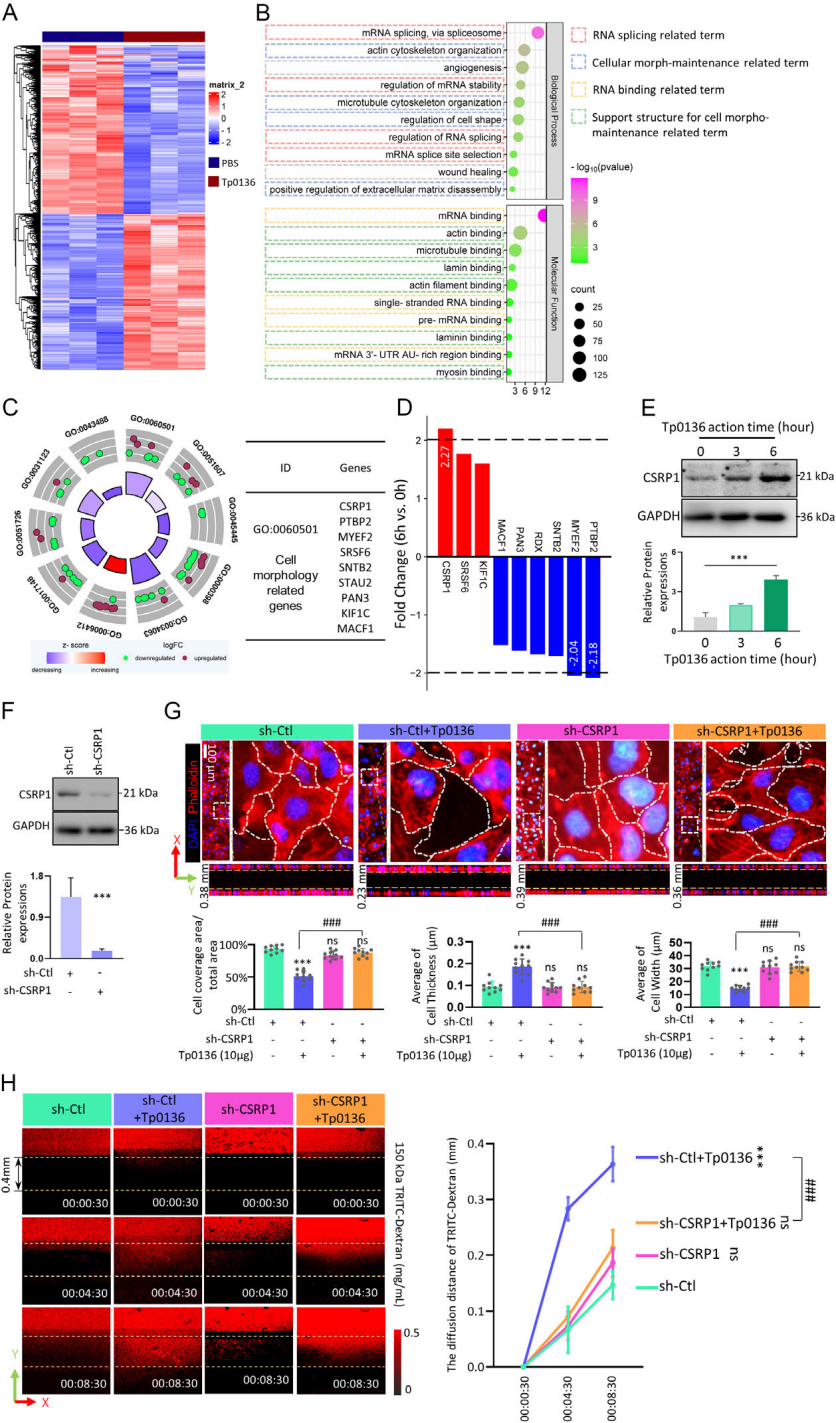
Tp0136 upregulated the expression of CSRP1 to enlarge the intercellular junctions in HMEC‐1 cell line. A) Heatmap of the normalized deviations of expression for a total of 5,457 differentially expressed genes (enriched by *P* < 0.001). B) GO analysis (GOTERM_BP_DIRECT category and GOTERM_MF_DIRECT category) for the overall differentially expressed genes. C) Gene ontology analysis of RBPs with different expression levels after Tp0136 protein treatment. Left: GO circle for the function annotation of the 146 RBPs; Right: List of the information involved in GO:0 060 501. D) Fold changes and qPCR validation of 9 cell morphology maintenance RBPs. E) The protein level of CSRP1 during the Tp0136 treatment in HMEC‐1 cell line. The values represent the mean ± SD of triplicates and are representative of three independent experiments. ***, *P* < 0.001. F) The protein level of CSRP1 after CSRP1 targeted shRNA treatment. The values represent the mean ± SD of triplicates and are representative of three independent experiments. ***, *P* < 0.001. G) Immunofluorescent staining of cytoskeleton by Phalloidin to calculate the cell coverage area in X‐Y panel, and average cell thickness or cell width in X‐Z panel after shRNA treatment with or without Tp0136 addition. The values represent the mean ± SD of triplicates and are representative of three independent experiments. ***, *P* < 0.001, ###, *P* < 0.001, ns, *P* > 0.05. H) Effect of CSRP1 on Tp0136‐induced vascular permeability in 3D microfluidic angiogenesis system. Time is indicated in hour:minute:second.

In order to examine the effects of Tp0136 treatment on gene expression patterns of vascular endothelial cells, HMEC‐1 cells were treated with 10 μg of Tp0136 protein and PBS for a duration of 6 h; then, RNAs were extracted and subjected to high‐throughput transcriptome RNA sequencing. Subsequently, we conducted calculations from RNA sequencing data to ascertain the normalized deviations of expression for a total of 5,457 genes that exhibited significant differential expression (enriched by *P* < 0.001). The subsequent heatmaps effectively illustrated that the transcriptome from the cells in Tp0136 group displayed discernible patterns in comparison to the cells in PBS group for 6 h (**Figure** **2**A). To obtain a thorough comprehension of the functional consequences of these genes that were expressed differentially in response to Tp0136, we classified them into Gene Ontology (GO) categories utilizing the DAVID database (http://david.abcc.ncifcrf.gov/).^[^ ^24^ ^]^ The analysis revealed that these genes exhibited significant enrichment in Biological Process terms pertaining to RNA splicing and maintenance of cell morphology, as well as in Molecular Function terms associated with RNA binding and support structure for cell morphology maintenance (Figure 2B).

RNA‐binding proteins (RBPs) play a pivotal role in regulating alternative splicing events and are intricately linked to the preservation of cellular morphology, serving as crucial regulators of vascular barrier function.^[^ ^13^, ^25^, ^26^, ^27^, ^28^ ^]^ Following a 6‐h treatment of HMEC‐1 cells with 10 μg Tp0136 protein, a total of 146 RBPs with *P* < 0.001 and fold change ≥1.5 were identified. In order to enhance the precision of the functional clustering of the 146 RBPs that were sorted based on a significance level of *P* < 0.001 and a fold change exceeding 1.5 in the expression data, the utilization of the GO circle was used to analyze their distinct functions (Figure 2C, Left). Among these RBPs, 9 RBPs (CSRP1, PTBP2, MYEF2, SRSF6, SNTB2, SUAU2, PAN3, KIF1C, and MACF1) exhibited a high frequency of clustering specifically related to the maintenance of cell morphology (Figure 2C, Right). To identify the most functionally relevant RBPs, a sorting process based on fold changes was conducted on the 9 RBPs. Among the 9 RBPs, CSRP1, SRSF6, and KIF1C were found to be upregulated, while the remaining 6 RBPs were downregulated after Tp0136 treatment. Furthermore, only CSRP1, PTBP2, and MYEF2 exhibited expression fold changes greater than 2. CSRP1, which was considered to regulate the permeability of blood vessels,^[^ ^29^ ^]^ was demonstrated with the highest fold change (fold change = 2.27) among these 9 RBPs (Figure 2D), was then followed with the confirmation of the protein level, which showed a significant increasing along the 10 μg Tp0136 treatment for 6 h (Figure 2E, *P* < 0.001), and CSRP1 was subsequently selected for further investigations.

To clarify the relationship between CSRP1 and cell morphology, CSRP1 sequence specific shRNA was used to knock down CSRP1 in HMEC‐1 cells to generate the sh‐CSRP1 HMEC‐1 cell line, with the mock shRNA was used to generate sh‐Ctl HMEC‐1 cell line (Figure 2F). These cell lines were seeded in the 3D microfluidic angiogenesis system with Tp0136 protein for 4 days (PBS as control), and stained with phalloidin for cell morphology checking (Figure 2G). It showed that following the elimination of CSRP1, the cell coverage area in the X‐Y panel returned to 100%, despite the presence of Tp0136 protein, aligning with the coverage observed in the sh‐Ctl group. In contrast, the cell coverage area in the sh‐Ctl + Tp0136 group was limited to 50%. Additionally, in the X‐Z panel, compared to the spheroidized and loosely arranged cells in the sh‐Ctl + Tp0136 group, we observed that upon the knocked out CSRP1, the cells exhibited a flat and dense arrangement, even in the presence of Tp0136. Subsequent permeability assessments indicated a significant reduction (*P* < 0.001) in the permeability of HMEC‐1 cells following CSRP1 depletion, irrespective of the presence of the Tp0136 protein, when compared to sh‐Ctl HMEC‐1 cells treated with 10 μg of Tp0136 protein, and the permeability of HMEC‐1 cells post‐CSRP1 knockout reverted to levels comparable to those observed in sh‐Ctl HMEC‐1 cells (Figure 2H). These outcomes implied that Tp0136 upregulates the expression of CSRP1 to induces bulging of cellular morphology and results in enlarged intercellular gaps, ultimately leading to enhanced vascular permeability.

One‐way ANOVA was used for comparisons between multiple groups and for comparisons between two groups. The values represent the mean ± SD of triplicates and are representative of three independent experiments. ***, *P* < 0.001, ###, *P* < 0.001, ns, *P* > 0.05.

### 2.3 Tp0136 Mediated Alternative Splicing of Myosin X (MYO10) Through CSRP1

**Figure 3:**
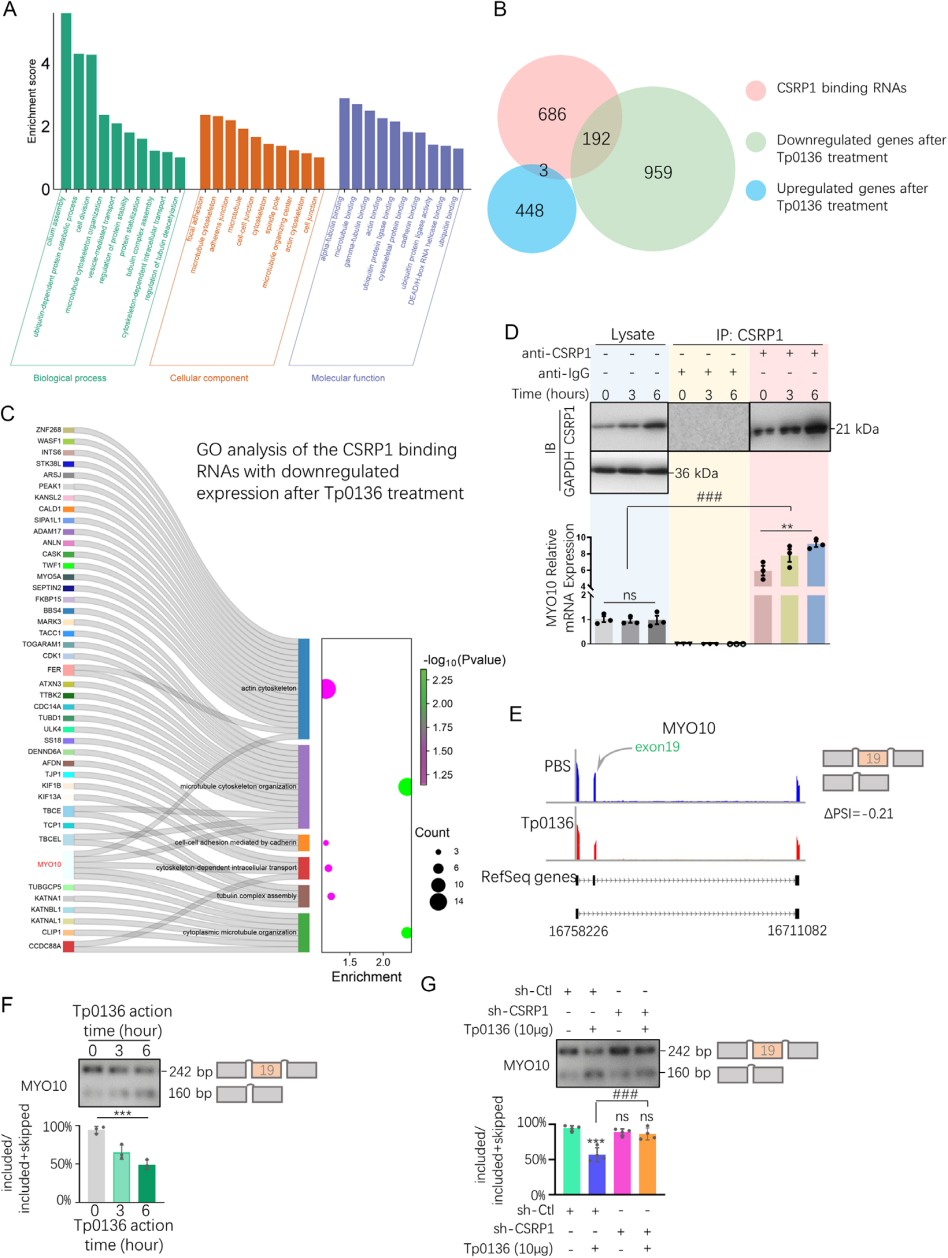
Tp0136 mediates alternative splicing of MYO10 through CSRP1. A) GO analysis of the 881 genes that enriched in both CSRP1 binding RNAs and skipping‐exon events. B) Venn diagram of CSRP1 binding RNAs in pink, downregulated genes after Tp0136 treatment in green, and upregulated genes after Tp0136 treatment in blue. C) Sankey map and GO bubble map depict the downregulated genes which were actin cytoskeleton related and were CSRP1 bind after Tp0136 treatment. D) CSRP1 protein‐MYO10 mRNA immunoprecipitation. The values represent the mean ± SD of triplicates and are representative of three independent experiments. ***, *P* < 0.001, ###, *P* < 0.001, ns, *P* > 0.05. E) MISO algorithm analysis of the MYO10 alternative splicing. F) Validation of the alternative splicing variants switch in MYO10 exon19 after Tp0136 protein treatment. Upper: Alternative splicing variants detection of MYO10 exon19 in high resolution agarose gel; Lower: Alternative splicing ratio of the MYO10 exon 19 calculated from high resolution agarose gel band grayscale. The values represent the mean ± SD of triplicates and are representative of three independent experiments. ***, *P* < 0.001. G) Validation of the alternative splicing variants switch in MYO10 exon19 in sh‐Ctl HMEC‐1 and sh‐CSRP1 HMEC‐1 cell lines with/without Tp0136 protein treatment. Upper: Alternative splicing variants detection of MYO10 exon19 in high resolution agarose gel; Lower: Alternative splicing ratio of the MYO10 exon 19 calculated from high resolution agarose gel band grayscale.

To investigate potential alternative splicing events associated with CSRP1, the proportion of alternative events influenced by CSRP1 was calculated from the RNA sequencing data firstly. Among the alternative splicing data for the skipping‐exon event, 881 genes were enriched by CSRP1. A comprehensive analysis revealed the identification of 881 genes, accounting for 28.2% of the total alternative splicing events. Afterwards, we conducted a GO analysis on 881 genes enriched with CSRP1 that were associated with skipping‐exon events. In the Biological Process category, these genes primarily cluster around terms pertaining to cell morphological support, such as cytoskeletal organization, and regulatory protein expression, including protein stabilization. These suggested that these genes with alternative splicing may be linked to the regulation of cell morphology through cytoskeleton protein expression. In the Cellular Component category, these genes were mainly enriched in terms like cell–cell junction and microtubule organization, indicating that these alternative splicing genes may aid in supporting cell morphology within cells through the internal skeleton and stretched to maintain cell morphology. The aggregation of terms such as cytoskeletal protein binding and other related terms in Molecular Function implies that genes with alternative splicing may be necessary for regulating cell morphology by binding to skeleton proteins in molecular biological functions (**Figure** **3**A). To clarify the expression levels of these genes enriched by CSRP1 that undergo skipping‐exon event and are closely related to cell morphology and cytoskeleton, a Venn diagram analysis was performed to compare the 881 genes (in pink) with those showing significant expression alterations in response to Tp0136 treatment. The genes downregulated by Tp0136 (downregulated genes after Tp0136 treatment: 1151 genes) were shown in green, and the genes upregulated (up regulated genes after Tp0136 treatment: 451 genes) were shown in blue. It revealed that 192 genes fulfilled the criteria of being enriched by CSRP1 and exhibiting a decrease in expression following Tp0136 treatment, whereas only 3 genes met the conditions of CSRP1 enrichment and demonstrated an increase in expression upon Tp0136 treatment (Figure 3B). Then, a GO analysis was conducted on the 192 genes, and 42 genes exhibiting cytoskeleton‐related terms were chosen for further Sankey analysis, and MYO10 was identified as the predominant protein present in the majority of terms associated with cytoskeletal organization (Figure 3C).

To confirm the interaction between the CSRP1 protein and MYO10 mRNA, RNA‐protein immunoprecipitation was used firstly. The findings demonstrated that CSRP1 protein was pulled down with beads covered with anti‐CSRP1 antibody and the level of enrichment increasing in correlation with the duration of Tp0136 protein exposure. Concurrently, we assessed the MYO10 mRNA released from CSRP1 protein by qPCR, and the results showed that MYO10 mRNA was pulled down in conjunction with the enrichment of CSRP1 protein (*P* < 0.001), with the enrichment levels of both MYO10 mRNA and CSRP1 protein exhibiting a corresponding increase as the duration of Tp0136 protein exposure was extended (*P* < 0.01, Figure 3D), confirming the CSRP1 protein binds to MYO10 mRNA. To elucidate the alternative splicing site of MYO10, a multiple‐input single‐output analysis was conducted on the alternative splicing data of MYO10, revealing that exon 19 underwent skipping following a 6‐h treatment with Tp0136 (Figure 3E). Subsequently, exon‐19 specific alternative splicing primers were used to confirm the alternative splicing pattern of MYO10 in the presence of Tp0136. The results demonstrated that with prolonged treatment of the Tp0136 protein, the inclusion rate of exon 19 included variant of MYO10 (included isoform: PCR product length 242 bp) among all alternative splicing variants (included+skipped) progressively declined from nearly 100% to 50%. Conversely, the exclusion rate of exon 19 in the MYO10 gene (skipped isoform: PCR product length 160 bp) correspondingly increased from less than 1% to ≈50% (*P* < 0.001, Figure 3F). To investigate the role of CSRP1 in the alternative splicing of MYO10 exon 19, an analysis was undertaken focusing on the splicing pattern of MYO10 exon 19 within the sh‐CSRP1 cell line. In comparison to the sh‐Ctl + Tp0136 group, where the exon 19 included variant of MYO10 constituted 50% of the total, the elimination of CSRP1 resulted in a significant increase in the proportion of the exon 19 included variant of MYO10 (*P* < 0.001), which approached or exceeded 80%, irrespective of the presence of the Tp0136 protein. This finding suggests that the CSRP1 elimination leads to a substantial enhancement of the exon 19 included variant of MYO10, while concurrently abrogating the Tp0136‐induced exon 19 skipped variant (Figure 3G). These results suggested that Tp0136 mediated the MYO10 exon 19 skipping through CSRP1.

One‐way ANOVA was used for comparisons between multiple groups and for comparisons between two groups. The values represent the mean ± SD of triplicates and are representative of three independent experiments. ***, *P* < 0.001, ###, *P* < 0.001, ns, *P* > 0.05.

### 2.4 Tp0136 Downregulated the Protein Level of MYO10 Through CSRP1‐Mediated Alternative Splicing to Enlarge the Intercellular Junctions, Resulting in the Enhancing Permeability

**Figure 4:**
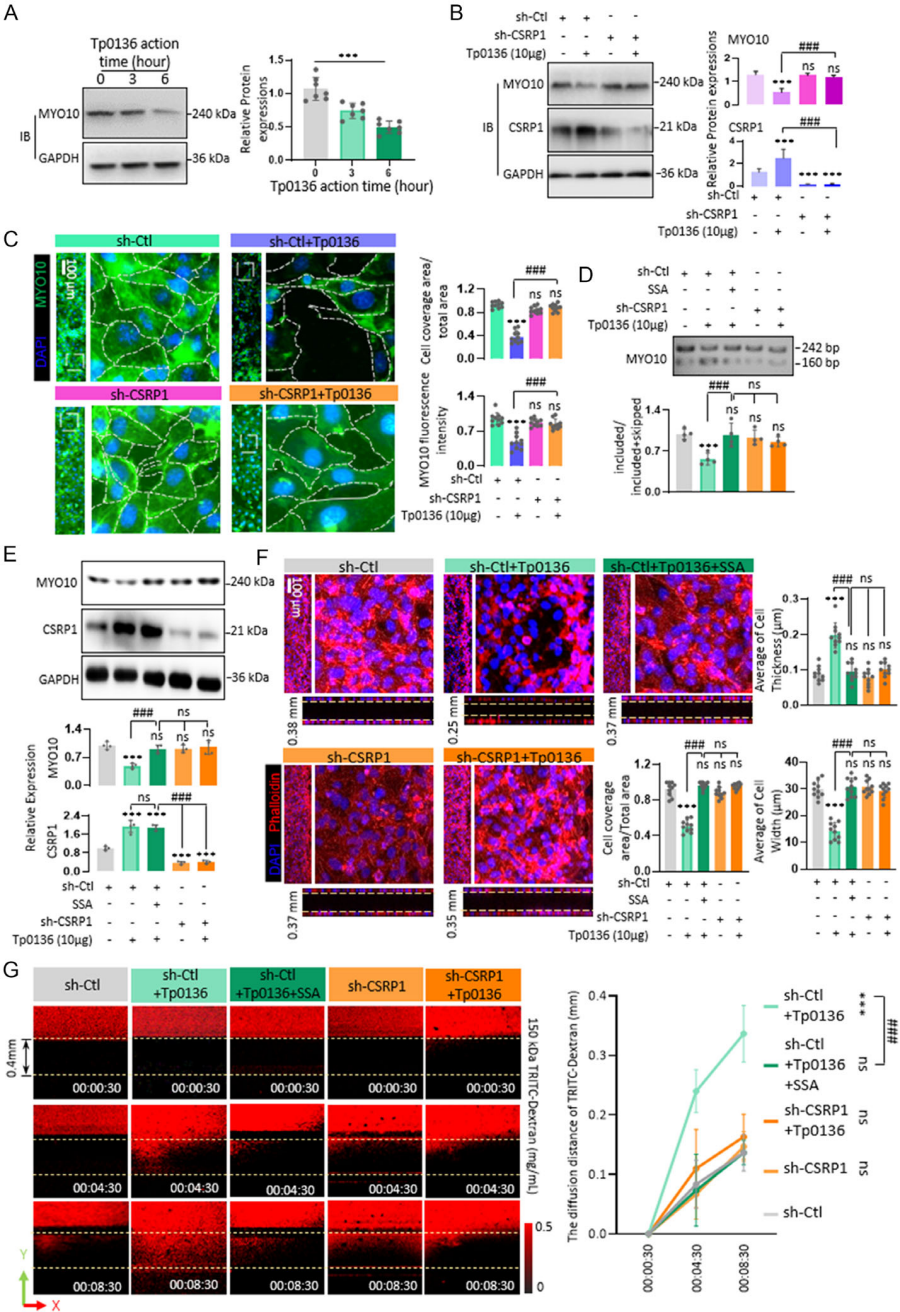
Tp0136 switched the alternative splicing pattern of MYO10 to downregulate the protein level through CSRP1, and enlarges the intercellular junctions. A) Protein level of MYO10 treated with Tp0136 protein. The values represent the mean ± SD of triplicates and are representative of three independent experiments. ***, *P* < 0.001. B) Protein levels of MYO10 and CSRP1 after CSRP1 knocked down and Tp0136 treatment. The values represent the mean ± SD of triplicates and are representative of three independent experiments. ***, *P* < 0.001. C) Photographs of the cells in the 3D microfluidic angiogenesis system to calculate the cell coverage and MYO10 fluorescence. The values represent the mean ± SD of triplicates and are representative of three independent experiments. ***, *P* < 0.001, ###, *P* < 0.001, ns, *P* > 0.05. D) Switch of MYO10 exon19 alternative splicing variants after alternative splicing inhibitor SSA treatment, Tp0136 protein treatment, and CSRP1 knock‐down. The values represent the mean ± SD of triplicates and are representative of three independent experiments. ***, *P* < 0.001, ###, *P* < 0.001, ns, *P* > 0.05. E) Protein levels of MYO10 and CSRP1 after SSA treatment, Tp0136 protein treatment, and CSRP1 knock‐down. The values represent the mean ± SD of triplicates and are representative of three independent experiments. ***, *P* < 0.001, ###, *P* < 0.001, ns, *P* > 0.05. F) Immunofluorescent staining of cytoskeleton by Phalloidin in the 3D microfluidic angiogenesis system to calculate the cell coverage area in X‐Y panel, and average cell thickness or cell width in X‐Z panel after SSA treatment with or without Tp0136 treatment. Scale bars = 100 μm. The values represent the mean ± SD of triplicates and are representative of three independent experiments. ***, *P* < 0.001, ###, *P* < 0.001, ns, *P* > 0.05. G) Effect of SSA on Tp0136‐induced vascular permeability in 3D microfluidic angiogenesis system. Time is indicated in hour:minute:second.

To corroborate the regulation of MYO10 expression by Tp0136 protein, the correlation between MYO10 protein expression and Tp0136 treatment duration was investigated in HMEC‐1 cell line. The expression of MYO10 was observed to decrease with the prolongation of Tp0136 protein treatment time (*P* < 0.001, **Figure** **4**A). Furthermore, the association between MYO10 protein expression patterns and those of CSRP1 protein was analyzed. It showed that upon silencing CSRP1, the expression level of MYO10 remained unaffected by Tp0136 (*P* > 0.05), and MYO10 expression reverted to the level observed in the sh‐Ctl group when compared to the sh‐Ctl+Tp0136 group (*P* < 0.001), suggesting that the expression pattern of MYO10 was inversely correlated with that of CSRP1 (Figure 4B). Immunofluorescence staining with MYO10 in a 3D microfluidic angiogenesis system revealed an enhancement in the expression of MYO10 (*P* < 0.001) accompanied with cell coverage area of HMEC‐1 cells (*P* < 0.001) upon the knockdown of CSRP1 compared to those in sh‐Ctl + Tp0136 group (Figure 4C), providing further evidence that the expression of MYO10 and cell coverage area were modulated by the expression of CSRP1.

Alternative splicing exhibits a close correlation with protein expression levels.^[^ ^30^, ^31^, ^32^, ^33^ ^]^ To verify that the downregulation of MYO10 expression under Tp0136 treatment is attributed to Tp0136‐mediated alternative splicing of MYO10 through CSRP1, alternative splicing inhibitor spliceostatin A (SSA, HY‐16 466)^[^ ^34^ ^]^ was used to attenuate the alternative splicing of MYO10. It revealed that the inhibitory effect of SSA on alternative splicing in HMEC‐1 mirrored that of knocked‐down CSRP1 in HMEC‐1 (*P* > 0.05), and Tp0136‐induced alternative splicing of MYO10 exon 19 was significantly suppressed (*P* < 0.001, Figure 4D). Then, the protein expression level was quantified, revealing an elevation in the expression of MYO10 protein following SSA treatment. This observation aligned with the expression pattern of MYO10 observed after CSRP1 knockdown, approaching the levels observed in the sh‐Ctl HMEC‐1 (Figure 4E). Then, phalloidin staining was used to visualize the morphological characteristics of HMEC‐1 cells treated with SSA. The results revealed that in sh‐Ctl HMIC‐1 cells, the application of SSA counteracted the spheroidization of cell morphologies and loose connections induced by Tp0136, which were analogous to those observed in sh‐Ctl, sh‐CSRP1, and sh‐CSRP1 + Tp0136 cells (Figure 4F). Furthermore, the cell coverage rate of sh‐Ctl HMEC‐1 cells following SSA treatment was significantly elevated compared to that following Tp0136 treatment (*P* < 0.001), approaching the level observed in sh‐Ctl HMEC‐1 cells, and the thickness of sh‐Ctl cells attached to the lumen, after concurrent treatment with Tp0136 and SSA, recovered to the level of untreated sh‐Ctl cells, resembling the cell thickness observed after CSRP1 knockout (*P* > 0.05, Figure 4F). Additionally, the permeability of the sh‐Ctl + Tp0136 + SSA group was significantly decreased when compared to that in the sh‐Ctl + Tp0136 group (*P* < 0.001) and return to the levels of sh‐Ctl group and the CSRP1 knockout group (*P* > 0.05, Figure 4G). This result further suggested that inhibiting MYO10 alternative splicing may aid in repairing the large cell gap induced by Tp0136 protein.

In summary, these findings suggest that Tp0136 elicited a reduction in MYO10 expression via CSRP1‐mediated alternative splicing, which subsequently results in the spheroidization of cell morphology and the enlargement of cell junction gaps.

One‐way ANOVA was used for comparisons between multiple groups and for comparisons between two groups. The values represent the mean ± SD of triplicates and are representative of three independent experiments. ***, *P* < 0.001, ###, *P* < 0.001, ns, *P* > 0.05.

## 3 Discussion

**Figure 5:**
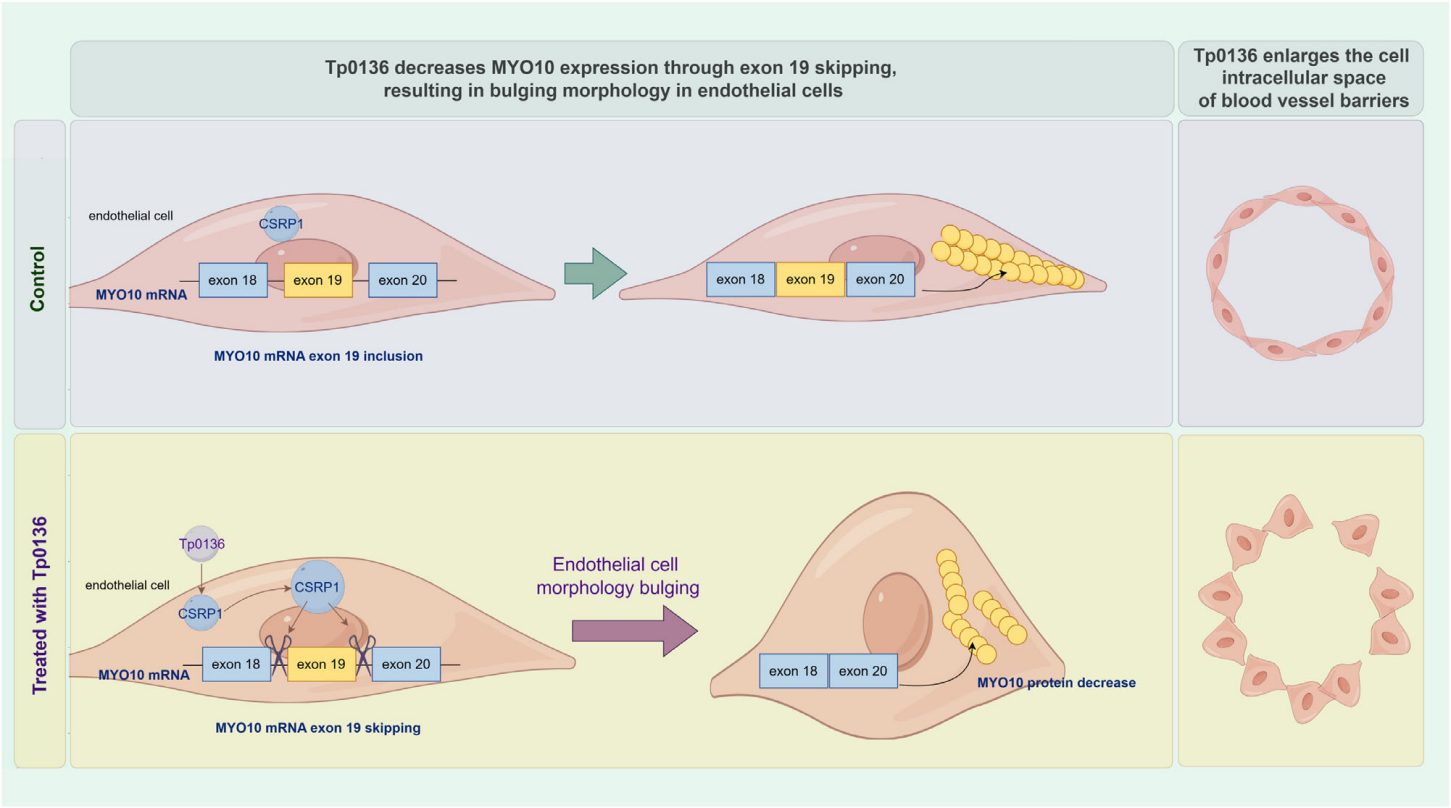
*Treponema pallidum* protein Tp0136 inhibits the expression of MYO10, leading to spheroidization of vascular endothelial cells and enlargement of intercellular gaps.

Investigating the mechanism underlying the dissemination of *Treponema pallidum* from the wounds to other organs serves as the foundation for comprehending its pathogenic mechanism and subsequently blocking its dissemination. Our previous study utilizing the syphilis rabbit model has demonstrated that Tp0136 facilitated the dissemination of *Treponema pallidum* from the primary chancres to distal organs. Furthermore, investigations using HMEC‐1 cells have revealed that Tp0136 promotes angiogenesis by activating the PI3K‐AKT signaling pathway in vascular endothelial cells, ultimately leading to increased vascular permeability.^[^ ^5^ ^]^ However, the precise mechanism through which Tp0136 facilitates angiogenesis, ultimately resulting in increased vascular permeability, remains elusive. Utilizing the established in vivo study system and Tp0136 protein treatment protocol,^[^ ^5^ ^]^ we observed the primarily characterized by blood vessel endothelial cell spheroidization and enlargement of intercellular spaces. In the in vitro experiments, high‐throughput transcriptional sequencing in HMEC‐1 cell line analysis revealed a downregulation of MYO10 expression and an upregulation of CSRP1 upon Tp0136 treatment. Additionally, alternative splicing analysis demonstrated an increased skipping frequency of MYO10 exon 19 ruled by CSRP1 under the treatment of Tp0136. Furthermore, by inhibiting the alternative splicing of MYO10 exon 19 using splicing inhibitors, or knocking down CSRP1, we observed an increase in MYO10 expression, leading to the restoration of the morphology and intercellular connectivity of vascular endothelial cells to that observed in the negative control groups. These findings suggest that Tp0136 regulates MYO10 alternative splicing through CSRP1, resulting in the downregulation of MYO10 expression, which in turn leads to spheroidization in vascular endothelial cells, enlargement of intercellular spaces, and enhanced vascular permeability (**Figure** **5**).

Blood barrier endothelial cell morphological alterations have been reported as a supporting channel with enhanced permeability, facilitating the dissemination of pathogens and tumor cells.^[^ ^22^, ^35^, ^36^ ^]^ The cytoskeleton–myosin system serves as a pivotal factor in maintaining the morphology and barrier function of vascular endothelial cells, and both are indispensable.^[^ ^37^, ^38^ ^]^ Myosin, as a cellular component responsible for regulating cytoskeletal dynamics, is highly susceptible to alterations during the pathogenesis of various pathogens.^[^ ^39^ ^]^ It is reported that *Streptococcus pyogenes*, *Mycobacterium tuberculosis*, and *Mycobacterium ulcerans* regulate myosin assembly to alter cell morphology, thereby assisting pathogens to enter the bloodstream.^[^ ^40^, ^41^, ^42^ ^]^ Given the analogous pathogenicity between *Treponema pallidum* and the aforementioned bacteria, *Treponema pallidum* has been proven to promote the migration of cells in the blood vessels,^[^ ^43^, ^44^ ^]^ which indirectly indicates that *Treponema pallidum* also has the effect of regulating the cell morphology. The findings in this study, along with those of our previous article,^[^ ^5^ ^]^ collectively demonstrate the diverse morphological manifestations of vascular endothelial cytopathia induced by *Treponema pallidum* infection, from the perspective of the membrane protein Tp0136. These manifestations include excessive angiogenesis and cellular morphological changes characterized by spheroidization, leading to enlarged intercellular junctions. Our findings provide deepen insights into the mechanism by which *Treponema pallidum* facilitates its dissemination via the membrane protein Tp0136.

Alternative splicing serves as a crucial regulatory mechanism in governing the morphology and permeability of vascular endothelial cells,^[^ ^12^, ^45^, ^46^, ^47^ ^]^ and it has been identified as a crucial factor in the dissemination of infectious diseases.^[^ ^48^, ^49^ ^]^ In the investigation of the pathogenic mechanisms of *Treponema pallidum*, the alternative isoforms of XBP‐1 have been observed in response to stimuli from *Treponema pallidum* and facilitated the immune evasion.^[^ ^50^ ^]^ However, the regulatory mechanisms governing these alterations remain primarily confined to the IRE1α pathway linked to the endoplasmic reticulum stress response.^[^ ^50^, ^51^ ^]^ To the best of our knowledge, in the field of *Treponema pallidum* infection, there is no report on the alternative splicing that regulates the structure of vascular endothelial cells to participate in the infection and dissemination process. In this study, we investigated the effect of *Treponema pallidum* on vascular endothelial cells and its underlying molecular mechanism, with particular emphasis on elucidating the mechanism by which *Treponema pallidum* protein regulates the expression of myosin proteins through alternative splicing, ultimately enhancing the permeability of the vascular endothelial barrier.

CSRP1 localized in various cell types. Previous research has demonstrated that CSRP1 operates in conjunction with the cytoskeleton, particularly in the formation of mature focal adhesions between cells, where a reduction in its abundance is often necessary.^[^ ^52^ ^]^ Furthermore, studies have indicated that during focal inflammation, tissue cells at the wound site, including vascular endothelial cells, require substantial recruitment of myosin to facilitate the re‐establishment of strong adhesions. CSRP1 is implicated throughout this entire process.^[^ ^52^, ^53^ ^]^ In the process of infection and spread of pathogenic microorganisms, the regulatory effect of CSRP1 has not been found in mammalian models, but in the immunological studies of model animals such as fish and shellfish, it has been found that high expression of CSRP1 promotes the infection of pathogenic microorganisms,^[^ ^54^ ^]^ which provides a way to study the function of mammalian CSRP1 protein. As an RNA‐binding protein, CSRP1 is postulated to play a role in the process of alternative splicing.^[^ ^55^ ^]^ However, aside from our study demonstrating that CSRP1 regulates cell morphology through the modulation of myosin alternative splicing, there appears to be a paucity of research investigating the role of CSRP1 in regulating cellular function via alternative splicing.

As a member of the myosin family, MYO10 is widely recognized, similar to other myosins, for its role in regulating the mobility of cytoskeletal proteins and consequently influencing cell morphology.^[^ ^56^ ^]^ Deficiency in MYO10 can significantly impair the maintenance and function of cell morphology, potentially resulting in cell death.^[^ ^57^, ^58^ ^]^ In the context of infectious diseases, numerous studies have demonstrated that the integrity of the MYO10 gene and its protein expression can be compromised by pathogenic bacteria, facilitating the dissemination of pathogens such as the *Shigella flexneri* ^[^ ^59^ ^]^ and *Marburg virus*.^[^ ^60^ ^]^ In our study, we discovered that *Treponema pallidum* facilitates the isoform switch of MYO10 via the Tp0136 protein. Specifically, the isoform lacking exon 19 fails to encode the functional protein, leading to a downregulation of MYO10 protein expression, which consequently results in a spherical cell morphology and assists *Treponema pallidum* dissemination.

Our findings collectively demonstrate that Tp0136‐induced endothelial spheroidization and junctional widening are mediated through the CSRP1‐MYO10 axis, providing a mechanistic basis for *Treponema pallidum* dissemination. Notably, the reversal of these phenotypes by CSRP1 knockdown or splicing inhibition suggests that targeting CSRP1 activity (e.g., via RNA interference or small‐molecule inhibitors) or stabilizing MYO10 expression (e.g., through mRNA delivery or proteasomal blockade) may serve as potential therapeutic strategies to reinforce vascular barrier integrity and limit bacterial spread. These approaches could complement existing anti‐Tp0136 antibody‐based interventions,^[^ ^5^ ^]^ offering a multi‐faceted strategy to counteract syphilis progression. Future studies exploring the synergy between CSRP1/MYO10‐targeted therapies and conventional antibiotics may further optimize clinical outcomes.

This study has several limitations. Firstly, *Treponema pallidum* is widely regarded as an extracellular bacterium, yet how it regulates intracellular CSRP1 through the outer membrane protein Tp0136 remains unexplored in our study. Our previous research has demonstrated that *Treponema pallidum* can be phagocytosed into immune cells, which allows the release of bacterial proteins into the intracellular space to exert their functions.^[^ ^61^ ^]^ However, in the early‐stage electron microscopy scans of this study, we did not observe *Treponema pallidum* entering the cells, although this does not necessarily mean that Tp0136 is not present intracellularly. Secondly, in our previous study, we observed that *Treponema pallidum* within the anti‐Tp0136 group was unable to disseminate from cutaneous lesions to the testes, attributable to the block effects of anti‐Tp0136 antibodies to Tp0136 protein on angiogenesis.^[^ ^5^ ^]^ Through a series of histochemical analyses and complementary methodologies, we consistently encountered challenges in detecting traces of blood vessels within the lesions of the anti‐Tp0136 group. This limitation hindered our ability to investigate the alterations in vascular endothelial cell transcripts associated with this group. Finally, based on our prior research indicating that Tp0136 facilitates angiogenesis and enhances vascular permeability, the precise location of Tp0136‐mediated MYO10 alternative splicing remains uncertain, whether it occurs within the newly formed blood vessels or within the preexisting vasculature at this juncture. Additionally, we acknowledge that the lack of in vivo genetic knockout models limits direct validation of the CSRP1‐MYO10 axis in live animals. While our in vitro knockdown and splicing inhibition experiments (Figure 2G,H and 4D–G) strongly support the functional role of CSRP1 and MYO10, the technical challenges of implementing CRISPR/Cas9 or shRNA‐based knockout approaches in the rabbit model—currently, the only syphilis pathology‐recapitulating system—precluded direct in vivo mechanistic verification. Future studies using localized shRNA delivery in rabbits will be critical to bridging this gap.

In summary, the present study elucidated the mechanism underlying the Tp0136 protein's facilitation of MYO10 exon 19 skipping via the upregulation of vascular endothelial cell CSRP1. This upregulation subsequently led to downregulation of MYO10 expression, inducing morphological spheroidization in vascular endothelial cells and ultimately resulting in loosened cell connectivity. These findings have the potential to further clarify the mechanism underlying the dissemination of *Treponema pallidum*.

*Treponema pallidum* protein Tp0136 upregulates the expression of CSRP1 to enhance the alternative splicing of MYO10 exon 19. Consequently, this process leads to a reduction in the level of MYO10 protein and mediates the widening of the intercellular space within blood vessel endothelial cells.

## 4 Conclusion

In this study, we elucidated a novel mechanism by which *Treponema pallidum* membrane protein Tp0136 facilitates bacterial dissemination through vascular endothelial barrier disruption. Combining in vivo syphilitic rabbit models and in vitro 3D microfluidic systems, we demonstrated that Tp0136 induces endothelial cell spheroidization and intercellular junction widening, leading to enhanced vascular permeability. High‐throughput RNA sequencing revealed that Tp0136 upregulates the RNA‐binding protein CSRP1, which modulates the alternative splicing of MYO10 exon 19, resulting in MYO10 downregulation. Functional validation confirmed that CSRP1 knockdown or inhibition of MYO10 splicing (via spliceostatin A) restores endothelial morphology and barrier integrity, directly linking the CSRP1‐MYO10 axis to Tp0136‐mediated vascular pathology. These findings provide the first evidence that *Treponema pallidum* exploits host RNA splicing machinery to manipulate cytoskeletal dynamics, offering a paradigm shift in understanding bacterial dissemination mechanisms. Therapeutically, targeting CSRP1 activity or restoring MYO10 expression presents a promising strategy to counteract vascular leakage and limit systemic spread of *Treponema pallidum*.

## 5 Experimental Section

5.1

5.1.1

##### Preparation of the Recombinant T. pallidum Membrane Protein Tp0136

Recombinant *T. pallidum* membrane protein Tp0136 was purified, and endotoxins were removed as described in a previous study.^[^ ^20^ ^]^ Briefly, the full‐length Tp0136 was directly cloned into the pEXP‐5‐CT‐TOPO vector; then, the recombined plasmid was inserted into *E. coli* BL21 strains and grown in Luri–Bertani medium supplied with 60 μg mL^−1^ ampicillin. Protein expression was added 0.5 mmol L^−1^ isopropyl‐B‐Dthiogalactopyranoside (IPTG) at 25 °C for 6 h. The *E. coli* inserted with Tp0136 genes were harvested by centrifugation (6000 g, 15 min, and 4 °C) and lysed by a sonicator with PBS augmented with 10 μg mL^−1^ lysozyme (Solarbio, Beijing, China). The lysate was collected by centrifugation (12 000 g, 20 min, and 4 °C). The pellet at the bottom of the tube was solubilized overnight in a buffer containing 8 mol L^−1^ urea and 0.1 mol L^−1^ Tris‐HCl (pH = 8.0) at 4 °C. Tp0136 recombinant proteins were purified from the pellet and eluted with PBS. Endotoxin contamination was removed using trace dynamic chromogenic limulus reagent (Bioendo, Xiamen, China) and was maintained at less than 0.05 endotoxin units (EUs)/mL. A bicinchoninic acid (BCA) (Takara Bio, Inc., Kusatu City, Japan) assay kit was used to estimate the concentrations.

##### Sample Preparation for Electron Microscopy

Samples were embedded using a protocol for scanning electron microscopy.^[^ ^62^, ^63^ ^]^ Briefly, after fixation, the samples were post‐fixed in 2% osmium tetroxide and 1.5% potassium ferricyanide v v^−1^ for 1 h on ice, incubated in 1% thiocarbohydrazide in dH_2_O w v^−1^ for 20 min, followed by 2% osmium tetroxide in dH_2_O w v^−1^ for 30 min, and then washed in dH_2_O and incubated overnight in 1% aqueous uranyl acetate at 4 °C. Cells were then stained with Walton's lead aspartate for 30 min at 60 °C. The coverslips were removed from the dishes after submerging the bottom in methanol for 20 min to soften the glue. The cells were then dehydrated stepwise through an ethanol series on ice, incubated in a tin foil container in a 1:1 propylene oxide and Durcupan resin mixture, and embedded in Durcupan ACM resin according to the manufacturer's instructions (Sigma‐Aldrich).

##### Cell Culture

Human microvascular endothelial cell line‐1 (HMEC‐1) (Procell Life Science & Technology, China) was maintained in HMEC‐1 cell‐specific medium (Procell Life Science & Technology, China) in a humidified incubator at 37 °C and 5% CO_2_ for 3 days, and the medium was changed every 24 h.

For the HMEC1 CSRP1 knock‐down cell line construction, 5 × 10^6^ HMEC1 were infected with lentivirus expressing short hairpin RNA (shRNA) targeting human CSRP1 gene (Santa Cruz Biotechnology, Dallas, TX, http://www.scbt.com) and selected with puromycin for 4 days; nontargeting viral particles were used as a control. The alternative splicing inhibitor SSA was added as described previously.^[^ ^64^ ^]^ Briefly, the final concentration of 100 ng mL^−1^ SSA was prewarmed with the HMEC1 culture medium and added to the 6‐well plate during medium change for a duration of 6 h.

To ensure the uniformity of cell fusion across experimental batches, a standardized cell inoculum (2 × 10^7^ cells/mL) was utilized, and a single pore cell from each batch was subjected to CD31 staining for cell‐edge determination. The boundaries of each cell were subsequently analyzed using the ImageJ software, with each cell being color‐coded. The extent of cell fusion was quantified by determining the ratio of the colored area within a given visual field to the total area of that field.

##### Immunocytochemistry

Cells were fixed in phosphate‐buffered saline (PBS) containing 4% paraformaldehyde (PFA) for 30 min at room temperature. Thereafter, all cells were blocked with 5% FBS and Triton X‐100 and incubated with the MYO10 primary antibody (Santa Cruz, USA). The cells were then rinsed with PBS and incubated with species‐specific Alexa Fluor 488‐conjugated secondary antibodies (Thermo Fisher, USA), followed by staining with DAPI (Sigma‐Aldrich, USA) to counterstain the nuclei.

##### 3D Microfluidic Angiogenesis System and Detection of Vascular Permeability

As previously described, angiogenesis analysis was based on a microfluidic model and 3D cell culture to simulate angiogenesis in vivo (ref. to LW). HMEC‐1 were dissociated, pelleted, and suspended in a concentration of 2 × 10^7^ cells mL^−1^. Two microliters of the cell suspension was dispensed into the perfusion inlet and incubated for 10 days. When the cells formed a stable monolayer, stimulation factors were added to the bottom perfusion channel, which was then placed in a shaker for continuous perfusion. On day 10 after stimulation, 50 mL of 150 kDa TRITC‐Dextran solution (Sigma‐Aldrich, USA) was added to the perfusion channel inlet and time‐lapse images were acquired at 30‐s intervals using a confocal microscope. The cytoskeleton and nuclei were stained via immunofluorescence, using a Phalloidin‐iFluor 555 reagent (Abcam, UK) and an antifluorescence quenching blocking solution containing DAPI (Beyotime, China), respectively. A laser confocal scanning microscope (LSM780, Zeiss, Germany) was used to obtain 3D scanning images.

##### Cell Number Counting by Flow Cytometry and DAPI Number Calculation

To quantify the HMEC‐1 cell population within the microfluidic system, the cells were enzymatically dissociated into a single‐cell suspension using 50 μL of a Trypsin containing 0.05% EDTA. The enzymatic reaction was subsequently halted by the addition of an equal volume of culture medium. Following the removal of the supernatant, the cells were resuspended in PBS, stained with CD31‐FITC, and enumerated using flow cytometry.

To ascertain the cell count within a 100 × 100 μm area of the microfluidic system, we used a visual enumeration method using DAPI staining. This procedure involved selecting and analyzing 10 distinct fields of view.

##### Cell Assessment Validation Through CCK‐8 Assay

Total cell viability was examined using cell counting kit‐8 (CCK‐8) assay. The samples were processed according to the protocol of the CCK‐8 assay kit (Beyotime, Shanghai, China) before determination of absorbance on a reader. After subtracting the absorbance of the blank control, the absorbance values of the samples were used to calculate the total cell viability rate.

##### RNA‐Sequencing Data Analysis and Visualization

RNA sequencing data were analyzed and visualized based on the methodology reported in a previous study.^[^ ^65^ ^]^ Paired‐end sequencing reads were trimmed, and quality was checked using Fastqc (version 0.11.7). Alignment to the human genome was performed using STAR software (version 2.5.2a). Differential gene expression analysis was performed using the RStudio software (version 1.0.153). For the study of differentially expressed genes exposed to the Tp0136 recombinant protein, we conducted a comparative analysis between the group treated with the Tp0136 recombinant protein and the untreated group, identifying a gene subset with a significance level of *P* < 0.001. To identify genes of greater research significance and more pronounced changes, those with a *P* < 0.001 and a gene fold change ratio ≥1.5 following treatment with Tp0136 recombinant protein were selected as the target gene set. Subsequent sequencing based on the change ratio resulted in the selection of the top 100 genes for further analysis using Gene Ontology (GO) and Kyoto Encyclopedia of Genes and Genomes (KEGG) pathways. The GO and enrichment analyses of RNA sequencing data were conducted using Panther (http://pantherdb.org/) and Enrichr (https://amp.pharm.mssm.edu/Enrichr/) on specified gene lists. To more clearly observe the enriched function term of differentially expressed genes, we filtered the function term with a significance level of *P* < 0.001 in the GO enrichment analysis. In conducting differentially expressed gene pathway enrichment analysis, we used a methodology similar to that utilized in GO analysis to identify the genes exhibiting the most significant changes. Subsequently, these identified genes were subjected to analysis using the KEGG database.

The splice junctions were identified using TopHat. Uniquely mapped reads (75–84% of sequenced reads) were selected for coverage and junction read count assembly. Replicate MATS (rMATS) was used to analyze alternative splicing events.^[^ ^66^ ^]^ The raw sequence data reported in this article have been deposited in the Genome Sequence Archive (Genomics, Proteomics & Bioinformatics 2021) in the National Genomics Data Center (Nucleic Acids Res 2022), China National Center for Bioinformation/Beijing Institute of Genomics, Chinese Academy of Sciences (GSA‐Human: HRA005158), which are publicly accessible at https://ngdc.cncb.ac.cn/gsa‐human.

##### Western Blotting

Western blotting was performed as previously described.^[^ ^67^ ^]^ The information and dilutions of CSRP1, MYO10, and GAPDH antibodies can be found in Supporting Information. Bands were visualized using the Gel Recording System (Bio‐Rad, USA) or iBright CL750 Imaging System (Thermo Fisher Scientific, USA).

##### RNA‐Protein Immunoprecipitation

RNA immunoprecipitation and RIP‐PCR was performed following the protocol described previously.^[^ ^68^ ^]^ Briefly, cells cultured in 10 cm plates were washed twice with ice‐cold PBS and scraped off in 1 mL PBS. Then, the cell was centrifuged and resuspended in an equal pellet volume of complete RIP lysis buffer (Merck Millipore). Five micrograms of antibody was prebound to protein A/G magnetic beads in an immunoprecipitation buffer (20 mM Tris‐HCl pH 7.5, 140 mM NaCl, 0.05% TritonX‐100) for 2 h and then incubated with 100 μL cell lysates over night at 4 °C with rotation. Then, RNA was eluted from the beads by incubating with 400 μL elution buffer for 2 h. The eluted RNA was precipitated with ethanol and dissolved with RNase‐free water. Enrichment of certain fragments was determined by real‐time PCR. The primer used in RIP‐PCR is listed in *Supporting Information*.

##### Experimental of Alternative Splicing

Regarding the screening of alternative splicing candidates associated with CSRP1 binding, the RNA‐Protein Interaction Prediction (RPISeq) database (Iowa State University, http://pridb.gdcb.iastate.edu/RPISeq/) was utilized and followed with TopHat analysis to identify junction sites, thereby obtaining candidate genes for CSRP1‐specific alternative splicing. Specific primers flanking predicted sites of alternative splicing were used for RT‐PCR amplifications. PCR products were separated by high‐resolution agarose gel electrophoresis. The primer used in alternative splicing detection is listed in *Supporting Information*.

##### Statistical Analysis

All experiments were performed at least three times, and the data were presented as mean ± standard deviation. GraphPad Prism 8.0 (GraphPad Software, La Jolla, CA, USA) was used for all the statistical analyses. Student's *t* test or nonparametric tests were used when two groups were compared. One‐way analysis of variance (ANOVA) was used to compare the mean values of three or more groups with one independent variable. Two‐way ANOVA was used to compare the mean values three or more groups with two independent variables. Post hoc comparisons were performed using Tukey's test. Statistical significance was defined as a two‐sided *P* value of  <0.05.

##### Data Availability

The RNA‐seq sequencing data reported in this article have been deposited in the Genome Sequence Archive (Genomics, Proteomics & Bioinformatics 2021) in the National Genomics Data Center (Nucleic Acids Res 2022), China National Center for Bioinformation/Beijing Institute of Genomics, Chinese Academy of Sciences (GSA‐Human: HRA005158), which are publicly accessible at https://ngdc.cncb.ac.cn/gsa‐human. Source data are provided with this article. The raw immunofluorescence and statistical figures can be obtained on the figshare website, DOI: 10.6084/m9.figshare.26 197 763.

## Conflict of Interest

The authors declare no conflict of interest.

## Author Contributions

**Yu Lin**: conceptualization (equal); methodology (lead); project administration (lead); resources (equal); software (lead); supervision (equal); validation (lead); visualization (lead); writing—original draft (lead); writing—review editing (lead). **Xi Luo**: conceptualization (equal); methodology (equal); project administration (equal); validation (equal); visualization (equal). **Xu Shen**: resources (equal); software (equal); validation (supporting). **Xiao‐Lin Fu**: methodology (supporting); validation (supporting); visualization (supporting). **Li‐Rong Lin**: funding acquisition (equal); resources (equal); visualization (equal). **Tian‐Ci Yang**: conceptualization (lead); resources (lead); supervision (lead).

## Supporting information

Supplementary Material

## Data Availability

Data sharing is not applicable to this article as no new data were created or analyzed in this study.
