# Supplementary material for: Treponema pallidum Protein Tp0136 Induces Spheroidization of Vascular Endothelial Cells, Resulting in Widened Intercellular Junctions and Enhanced Vascular Permeability
Source: Small Sci. 2025 May 6;5(7):2500046. doi: 10.1002/smsc.202500046 (PMC12257882; doi:10.1002/smsc.202500046)
Supplement: Supplementary file 1 — Supplementary Material [file SMSC-5-2500046-s001.pdf]

Treponema pallidum protein Tp0136 Induces Spheroidization of Vascular Endothelial Cells, Resulting in Widened intercellular junctions and Enhanced Vascular Permeability

Supporting Information

*Yu Lin, Xi Luo, Xu Shen, Xiao-Lin Fu, Li-Rong Lin, Tian-Ci Yang**

1. **Primers for qRT-PCR**

Gene symbol	Forward	Reverse	Production Length
MYO10	ATCCTGTGTTGAACGAGCT ATT C	CCCAAAGCGACTAGAGTT GTT G	97 bp

2. Primers for alternative splicing detection

Splicing Site	Forward	Reverse	Production Length
MYO10 Exon 19	GAACATGTTTCAAGCCGCA ACAACC	CGGATTCTCAGTCTCCA GCATC	Full length: 242 bp Skipped length: 160 bp

3. Knockdown Assay Information

Name	Supplier	Catalogue No.	Resistance Gene
sh-CSRP1	Santa Cruz	sc-45367-V	Puromycin
sh-Ctl	Santa Cruz	sc-108080	Puromycin

4. Antibodies and chemicals for Western Blot or immunostaining

Antibody name	Supplier	Catalogue No.	Application & Dilution
CSRP1	abcam	ab70010	For Western Blot: 1:1000
MYO10	Santa Cruz	sc-166720	For Western Blot: 1:1000<br>For Immunostaining: 1:100
GAPDH	abcam	ab8245	For Western Blot: 1:2000
Goat anti-Rabbit IgG	abcam	ab205718	For Western Blot: 1:5000
Rabbit anti-Mouse IgG	abcam	ab6728	For Western Blot: 1:5000
Goat anti-Mouse IgG (Alexa Fluor® 488)	Thermo Fisher	A-10680	For Immunostaining: 1:5000
Phalloidin -iFluor 555	abcam	ab176756	Non-dilution
CD-31-FITC	abcam	Ab33858	Non-dilution
DAPI	Beyotime	P0131	Non-dilution
